# Comparison of Jaw Mode and Field Width for Left-Breast Cancer Using TomoDirect Three-Dimensional Conformal Radiation Therapy: A Phantom Study

**DOI:** 10.3390/healthcare10122431

**Published:** 2022-12-02

**Authors:** Haneul Kim, Jaehong Jung, Hyunseo Jung, Jibeom Jeong, Dohwa Lee, Hyun-Woo Jeong, Youngjin Lee

**Affiliations:** 1 Department of Radiological Science, College of Health Science, Gachon University, 191, Hambakmoero, Yeonsu-gu, Incheon 21936, Republic of Korea; 2 Department of Radiation Oncology, College of Medicine, Soonchunhyang University Bucheon Hospital, 170, Jomaruro, Bucheon-si 14584, Republic of Korea; 3 Department of Biomedical Engineering, Eulji University, 553, Sanseong-daero, Sujeong-gu, Seongnam-si 13135, Republic of Korea

**Keywords:** breast cancer, radiation therapy, TomoDirect (TD), three-dimensional conformal radiation therapy (3D-CRT), jaw mode and field width

## Abstract

It is very important to use effective parameters in the treatment plan of breast cancer patients in TomoDirect (TD)-three-dimensional conformal radiation therapy (TD-3DCRT). The objective of this study was to compare the radiation treatment plans to the parameters (jaw width and jaw mode) of TD-3DCRT for left-breast cancer. This study was conducted using the phantom, the jaw mode (fixed and dynamic) and field width (2.5 cm and 5.0 cm) were controlled to compare the TD-3DCRT treatment plans. There was small difference in the conformity index (CI) and homogeneity index (HI) values for target according to the jaw mode for each field width. As a result of observation in terms of dose, treatment time and unnecessary damage to surrounding normal organs could be minimized when dynamic jaw with a field width of 5.0 cm was used. In conclusion, we verified that the use of dynamic jaws and 5.0 cm field width was effective in left-breast cancer radiotherapy plan using TD-3DCRT.

## 1. Introduction

Radiation therapy for breast cancer treatment used various techniques such as intensity-modulated radiation therapy (IMRT), volumetric modulated arc therapy (VMAT), and three-dimensional conformal radiation therapy (3D-CRT). IMRT has been used to sufficiently address the target while reducing dose to the surrounding normal tissues. This is achieved by controlling the intensity of radiation using a multi-leaf collimator (MLC) [1]. In VMAT, the gantry head of linear accelerator rotates 360° around the patient and delivers radiation continuously. In addition, by using the MLC, the radiation intensity and radiation field are adjusted to treat tumors accurately while evading normal tissues [2,3]. According to Srivastava et al., although VMAT shortens the treatment time, it increases the lower radiation dose volume. In addition, it should be considered that a leakage dose is generated by the MLC used in VMAT, which affects the patient [4,5].

3D-CRT with a fixed beam for breast cancer patients could be reduce the excess dose to the surrounding normal tissues such as the heart, lung, contralateral breast, and esophagus [6,7]. In other words, it minimizes the radiation dose in normal organs as it can be delivered on multiple planes using more beams than in conventional radiation therapy. 3D-CRT is effective in protecting the heart because it involves a lower exposure dose than IMRT or VMAT. Aras et al. [8] reported that the mean absorbed dose to the heart with 3D-CRT is approximately 29.93% lower than that with IMRT. Being amenable to further refinements to maximize the heart sparing in left breast cancer irradiation, 3D-CRT is the most preferred and debated RT technique among the radiation oncologists treating these patient cohorts [9,10]. The dose-volume histogram (DVH) can verify quantitative assessment of the absorbed dose for the target and critical organs, and biological indexes such as the tumor control probability (TCP) and normal tissue complication probability (NTCP) also can predict the likelihood of radiation-induced complications in normal tissues and the local control rate of tumors [11,12,13].

TomoDirect (TD) uses the fixed beam on a Tomotherapy (Accuray Inc., Sunnyvale, CA, USA) that enables IMRT option as well as 3D-CRT including different jaw modes (fixed or dynamic jaw mode). The objective of this study was to compare the radiation treatment plans parameters (jaw mode and jaw width) of 3D-CRT for left-breast cancer using TD.

## 2. Materials and Methods

### 2.1. Phantom and CT Scan Images

**Figure 1:**
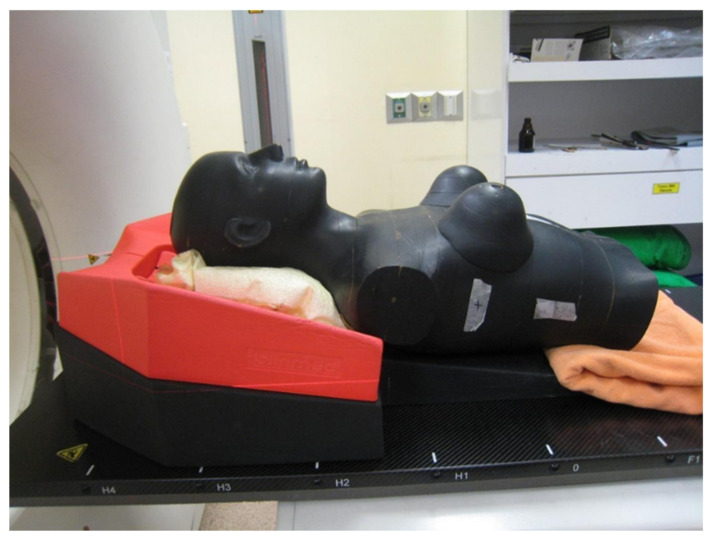
Female RANDO^®^ human phantom with tilted wedge immobilization devices and vac-lock.

A RANDO^®^ human phantom (Alderson Laboratory, New York, NY, USA) was used to implement the left-breast cancer treatment plan (Figure 1). It is an adult female phantom with a height of 175 cm and weight of 73.5 kg. A tilted wedge immobilization device at an angle of 15° was used. CT images for treatment planning were acquired using a CT simulator (SOMATOM Confidence, Siemens, Munich, Germany) with a slice thickness of 3 mm.

### 2.2. Treatment Planning

The planning target volume (PTV) and organs at risks (OARs) were contoured in MIM Maestro (MIM Software, Cleveland, OH, USA). All treatment structures including regions of interest (ROIs) and CT images were exported to an Accuray Precision (ver. 2.0.1.0) treatment planning system.

**Figure 2:**
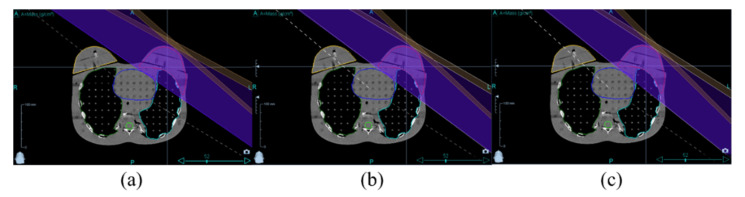
Example of TD plans in two directions with RAO {(**a**) 308° (**b**) 310° (**c**) 312°)} and LPO {(**a**) 121° (**b**) 123° (**c**) 125°)} in axial CT images with ROIs (PTV and OARs) for left-breast cancer.

**Figure 3:**
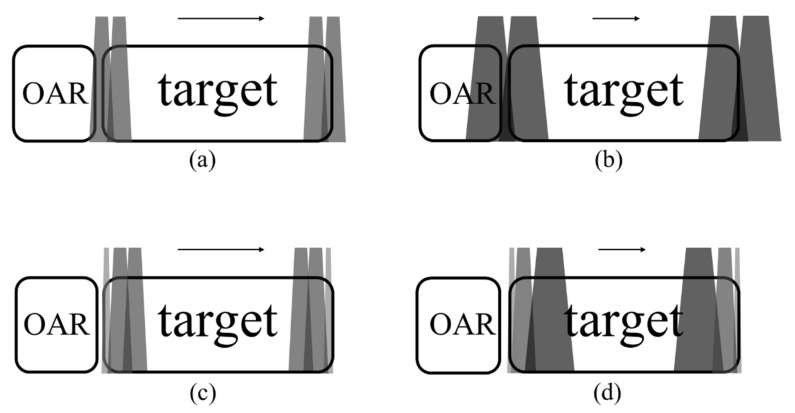
Illustrations of beam size of fixed jaw with field width of (**a**) 2.5 cm and (**b**) 5.0 cm, and dynamic jaw width with field width of (**c**) 2.5 cm and (**d**) 5.0 cm. Fixed mode has higher dose penumbras to OARs adjacent to the target.

A total of thirteen plans with different gantry angles were established to reduce the deviation of TD-3DCRT for left-breast cancer. The irradiation angle was the right anterior oblique {RAO, range (307°~313°)} and the left posterior oblique {LPO, range (120°~126°)} and changed of intervals 0.5° for delivery beam directions (a total of fifty-two plans) (Figure 2). The treatment margin was applied to consider the movement of the target. Two binary-MLC leaves were opened (margin: 12.5 mm) to the exterior of the skin. The prescription dose for the PTV was 50.40 Gy in 28 fractions. In the Tomotherapy, two jaw modes (fixed or dynamic) were selected by a medical physicist for treatment planning (Figure 3). The fixed jaw mode had only one field width regardless of the tumor volume, and the dynamic jaw mode dynamically applied a fixed jaw width according to the tumor volume in the superior–inferior directions. The size of the beam, treatment time, and absorbed dose are varied according to the jaw modes [14]. Furthermore, field widths of 1.0, 2.5, and 5.0 cm could be used in both the jaw modes. The field width of 1.0 cm was not applied to the dynamic jaw mode.

### 2.3. Evaluation

The DVH was used to evaluate the dose for different jaw modes and field widths. It can quantitatively analyze the treatment plan results by applying the guidelines for dose and volume to targets and OARs [15]. The mean and standard deviation (SD) of the conformity index (CI) and homogeneity index (HI) for the target were obtained in treatment planning system that provides calculated dose. Here, a value of close to 1 for CI and HI index means better. In addition, the beam on time (sec) was calculated for all plans.

In this study, the OARs were the spinal cord, heart, right lung, left lung, whole lung, and contralateral breast (right breast). These were expected to be significantly exposed to radiation during left-breast cancer treatment. Each OAR was evaluated by calculating the maximum absorbed dose ($D_{\max}$) and mean absorbed dose ($D_{\mathrm{mean}}$).

## 3. Results and Discussion

### 3.1. Target Coverage

The mean and SD of the CI and HI of the PTV are presented in Table 1. For a field width of 2.5 cm, the CI was 1.58 ± 0.06 in the fixed jaw mode and 1.56 ± 0.06 in the dynamic jaw mode, respectively (difference percentage = 1.2%). The HI was 1.07 ± 0.02 and 1.07 ± 0.01 in the fixed and dynamic jaw modes, respectively (difference percentage = 0.1%). For a field width of 5.0 cm, the CI was 1.65 ± 0.06 and 1.61 ± 0.07 in the fixed and dynamic jaw modes, respectively (difference percentage = 2.1%). The HI was 1.07 ± 0.02 and 1.06 ± 0.01 in the fixed and dynamic jaw modes, respectively (difference percentage = 0.3%). Deviation of target coverage was small for different gantry angles.

**Figure 4:**
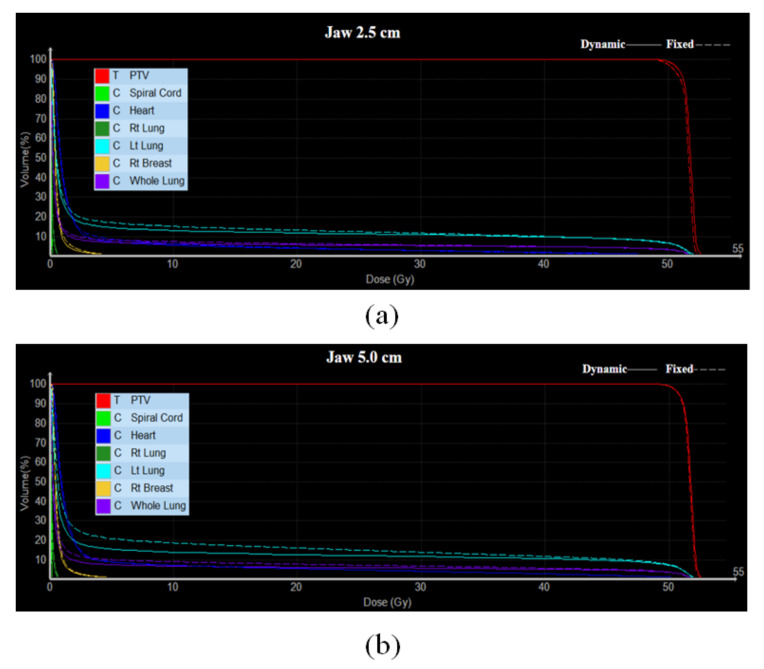
DVH comparison between fixed and dynamic modes with field width of (**a**) 2.5 cm and (**b**) 5.0 cm for TD-3DCRT plans {RAO (310°) and LPO (123°) directions}. As regards the left lung, using a field width of 5 cm produced a larger dosimetric difference between the fixed and dynamic jaw modes than a field width of 2.5 cm.

The CI values were close to each other (small difference) at a field width of 5.0 cm. Similarly, the difference in HI between the fixed and dynamic jaw modes was small. Overall, the target coverage was similar for different field width and jaw modes. Figure 4 shows the difference in calculated DVH of the PTV with OARs. The difference in CI and HI was low with respect to the adjustment of the parameters (field width and jaw mode) in this study. In addition, the maximum absorbed dose to the target should be ≤110% of the prescribed dose [16]. However, the differences of the beam on time were about 40% with respect to jaw modes (2.5 cm vs. 5.0 cm) in the dynamic modes.

The absorbed doses to the tumor and normal organs were varied by controlling the equipment variation factors using quantitative evaluation. The field width (2.5 cm and 5.0 cm) and jaw mode (fixed and dynamic jaw modes) were selected as the treatment plan parameters. A field width of 1.0 cm was excluded because it could only be applied in fixed jaw mode. TD-3DCRT establishes a treatment plan equivalent to tangential irradiation by setting two beams that are likely to be tangentially opposed beams, such as a linear accelerator (LINAC) in clinical practice.

### 3.2. OARs Dose

**Figure 5:**
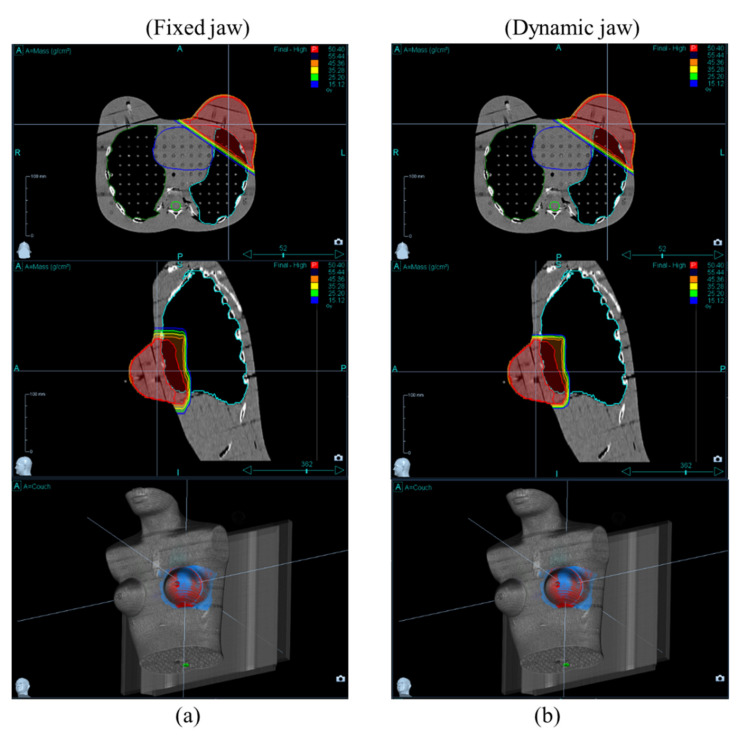
CT images (upper and middle rows) and 3D dose distribution (lower row) between jaw modes in (**a**) fixed and (**b**) dynamic jaw modes at the field width of 2.5 cm. The red and blue colors indicate target coverages of over 95% and 30% of the prescription dose, respectively.

**Figure 6:**
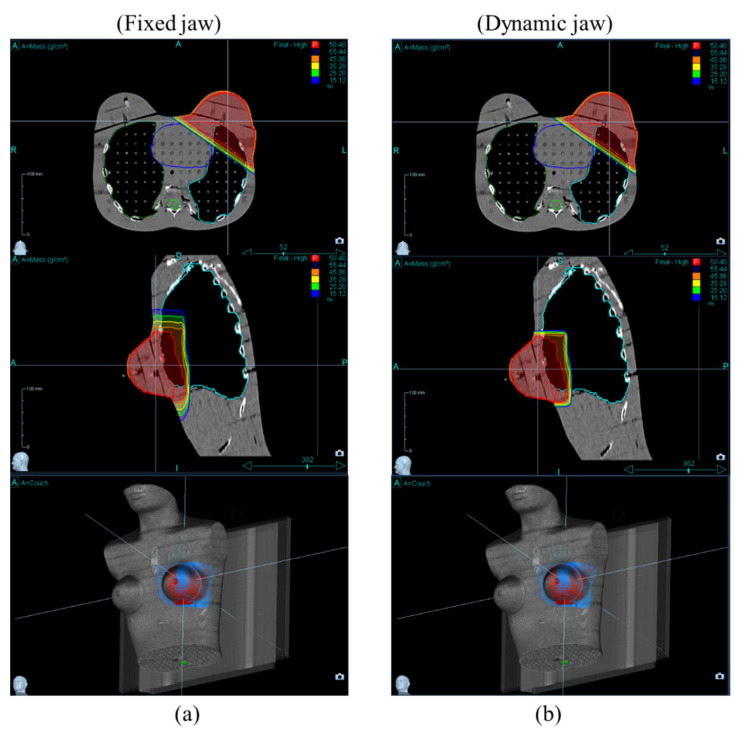
CT images (upper and middle rows) and 3D dose distribution (lower row) between jaw mode at (**a**) fixed and (**b**) dynamic jaw modes at the 5.0 cm field width. Red and blue colors indicate target coverage of more than 95% and 30%, respectively, of the prescription dose.

Figure 5 and Figure 6 show the dose distribution of the PTV and OARs at field widths of 2.5 cm and 5.0 cm, respectively. Table 2 shows the mean absorbed dose for the OARs for different field widths and jaw modes. In the radiation therapy of left-breast cancer, the ischaemic cardiovascular disease is of primary interest because that risk arises and persists several years after radiotherapy [17,18].

At a field width of 2.5 cm, the mean absorbed doses of the heart were 2.72 ± 0.29 Gy (maximum: 52.59 ± 0.38 Gy) and 2.69 ± 0.29 Gy (maximum: 52.64 ± 0.39 Gy) in the fixed and dynamic jaw modes, respectively. The values for the left lung were 6.62 ± 1.72 Gy (maximum: 53.80 ± 0.32 Gy) and 8.06 ± 0.30 Gy (maximum: 53.64 ± 0.32 Gy) in the fixed and dynamic jaw modes, respectively. The maximum absorbed doses of the spinal cord were 0.21 ± 0.01 Gy and 0.23 ± 0.01 Gy in the fixed and dynamic jaw modes, respectively.

At a field width of 5.0 cm, the mean absorbed doses of the heart were 3.43 ± 0.22 Gy (maximum: 52.28 ± 0.41 Gy) and 3.36 ± 0.22 Gy (maximum: 52.35 ± 0.41 Gy) in the fixed and dynamic jaw modes, respectively. Similarly, the values for the left lung were 8.06 ± 0.30 Gy (maximum: 53.64 ± 0.21 Gy) and 6.59 ± 0.34 Gy (maximum: 53.66 ± 0.27 Gy) in the fixed and dynamic jaw modes, respectively. The maximum absorbed doses of the spinal cord were 0.23 ± 0.01 Gy and 0.22 ± 0.01 Gy in the fixed and dynamic jaw modes, respectively.

The dose reduction in the dynamic jaw mode was verified through the results obtained for the mean absorbed doses of the OARs. The absorbed dose of the OARs in the dynamic jaw mode was lower than that in the fixed jaw mode regardless of the field width. At a field width of 2.5 cm, the mean absorbed dose in dynamic jaw mode for the heart and left lung decreased by approximately 1.1%, and 1.4%, respectively, compared with the fixed jaw mode. At a field width of 5.0 cm, the dynamic jaw mode for the heart and left lung decreased by approximately 2.1%, and 18.2%, respectively. Therefore, the dynamic jaw mode can reduce the probability of damage to OARs. The mean absorbed dose of the OARs was the lowest in the dynamic jaw mode with a field width of 2.5 cm. An increase in the field width yielded a difference according to the jaw mode (fixed and dynamic) for the left lung.

At a field width of 2.5 cm, the maximum absorbed doses in each organ were identical for the fixed and dynamic jaw modes, or marginally higher for the dynamic jaw. The absorbed dose for spinal cord decreased by approximately 2.2%, while for the heart and left lung increased by approximately 0.1%. At a field width of 5.0 cm, the maximum absorbed dose to the spinal cord decreased by approximately 7.2%. The maximum absorbed for heart increased by approximately 0.1%, and the left lung decreased by 0.2%. In general, the maximum absorbed doses of the OARs at a field width of 5.0 cm were higher in the dynamic jaw mode than in the fixed jaw mode except for the left lung. 

Sugie et al. [19] reported that the dynamic jaw is effective to achieve a good dose coverage in the breast and supraclavicular regional lymph nodes during breast cancer radiation therapy. In this study, the absorbed doses were compared mainly with the left lung, heart, and spinal cord among OARs around the target in the dynamic jaw mode during radiotherapy for left breast cancer. During radiotherapy with TD-3DCRT, it was verified that the dose was reduced by approximately 2.1% and 18.2% in the heart and the left lung, respectively, when the dynamic jaw mode had a 5.0 cm (rather than 2.5 cm) field width.

We could verify the OAR dose in the dynamic jaw at the field width of 5.0 cm under the conditions of this study. We consider that the results of this experiment would help to determine the optimal mode for 3D-CRT radiation treatment planning for patients with left-sided breast cancer in clinical practice. A dynamic jaw with a field width of 5.0 cm minimizes damage to the surrounding normal organs and reduces complications caused by radiation therapy with decreased the beam on time as about 40%. Therefore, we consider that our experimental result would effectively treat breast cancer which has the highest incidence in women among all types of cancer. However, the limitation of this study is that we used a phantom without clinical practice. A radiation treatment plan should consider that the result can vary because of the addition of patient-side variable factors such as the patient’s skin thickness and respiration pattern.

## 4. Conclusions

We compared the TD-3DCRT plans of left-breast cancer at different jaw modes and field widths. The difference in CI, HI, and maximum absorbed doses of the OARs between the fixed and dynamic jaw modes that small difference in this study. Therefore, the effectiveness of the dynamic jaw could not be verified. However, the mean absorbed doses of the OARs were lower in the dynamic jaw mode than in the fixed jaw mode regardless of the field width. To conclude, we verified that an optimum setting in a left-breast cancer radiotherapy plan using TD-3DCRT existed at a field width of 5.0 cm in the dynamic jaw mode. However, other parameters of TD-3DCRT should be considered according to patient-side parameters while planning breast cancer radiation treatment in clinical practice.

## Figures and Tables

**Table 1:** CI and HI of treatment target according to jaw mode (fixed and dynamic) and jaw width (2.5 cm and 5.0 cm).

	Field Width (2.5 cm)		Field Width (5.0 cm)	
Index	Fixed Jaw	Dynamic Jaw	Fixed Jaw	Dynamic Jaw
CI	1.58 ± 0.06	1.56 ± 0.06	1.65 ± 0.06	1.61 ± 0.07
HI	1.07 ± 0.02	1.07 ± 0.01	1.07 ± 0.02	1.06 ± 0.01
Beam on time (s)	111.27 ± 2.28	125.64 ± 1.03	67.01 ± 1.08	73.19 ± 0.97

**Table 2:** Mean absorbed dose (Gy) of the organ at risks (OARs) for two jaw modes (fixed versus dynamic) at a field width of 2.5 cm and 5.0 cm.

OAR	Field Width (2.5 cm)		Field Width (5.0 cm)	
	Fixed Jaw	Dynamic Jaw	Fixed Jaw	Dynamic Jaw
Heart	2.72 ± 0.29	2.69 ± 0.29	3.43 ± 0.22	3.36 ± 0.22
Right lung	0.18 ± 0.01	0.16 ± 0.00	0.21 ± 0.01	0.17 ± 0.01
Left lung	6.22 ± 1.72	6.13 ± 0.28	8.06 ± 0.30	6.59 ± 0.34
Right breast	0.61 ± 0.07	0.59 ± 0.06	0.69 ± 0.09	0.63 ± 0.08
Whole lungs	3.26 ± 0.12	2.99 ± 0.13	3.93 ± 0.14	3.21 ± 0.16

## Data Availability

Not applicable.
